# Differential Effects of Endocrine Therapy Type on Quality of Life in Older (≥70 Years) Women with Early-Stage Breast Cancer

**DOI:** 10.1245/s10434-024-16482-4

**Published:** 2025-04-26

**Authors:** Keva Li, Erin Moshier, Theresa Shao, Barry S. Rosenstein, Manjeet Chadha, REQUITE Consortium

**Affiliations:** 1 Department of Medical Education, Icahn School of Medicine at Mount Sinai, New York, NY USA; 2 Department of Population Health Science & Policy, Icahn School of Medicine at Mount Sinai, New York, NY USA; 3 Department of Hematology and Medical Oncology, Icahn School of Medicine at Mount Sinai, New York, NY USA; 4 Department of Radiation Oncology, Icahn School of Medicine at Mount Sinai, New York, NY USA

**Keywords:** Health-related quality of life, Patient-reported outcomes, Early-stage breast cancer, Estrogen receptor positive, Older women, Tamoxifen, Aromatase inhibitors, Endocrine therapy, Lumpectomy, Radiation therapy

## Abstract

**Background:**

There is limited data on health-related quality of life (HRQoL) in older breast cancer (BC) patients. This study examines patient-reported outcomes (PROs) by type of endocrine therapy (ET) prescribed, aromatase inhibitors (AI), or tamoxifen (Tam) to estrogen receptor-positive BC patients aged ≥70 years.

**Methods:**

This retrospective review includes 1052 women diagnosed with early-stage BC from the REQUITE study database, who underwent breast conservation surgery (BCS), and received adjuvant breast radiation therapy (RT), and ET as the only systemic therapy. Among them, 201 women were aged ≥70 years. The PROs were assessed by using EORTC-QLQ-C30, BR23, and Multidimensional Fatigue Inventory measures obtained at baseline after BCS, post-RT, and at 1, 2, and 3 years follow-up. Statistical analysis involves mixed model analysis of variance and propensity score weights.

**Results:**

Among the 201 women, 131 received AI, and 70 received Tam. The overall mean age of this cohort is 75.3 years. Compared with Tam, AI-treated patients experience worse insomnia and general and physical fatigue. Tam-treated patients experienced more physical and cognitive functioning decline than the AI-treated patients. The Tam-treated patients also reported more mental fatigue and reduced sexual enjoyment compared to the AI-treated patients.

**Conclusions:**

This study suggests a differential impact by type of ET on distinct HRQoL domains experienced by older postmenopausal women. Furthermore, larger prospective clinical trials are necessary to inform treatment decisions for older ER-positive BC patients, considering patient preferences and understanding trade-offs between disease outcomes and HRQoL.

The standard treatment for early-stage breast cancer (BC) in women has evolved over the years from mastectomy to breast conservation strategies that include multidisciplinary management.^1,2^ As per the National Comprehensive Cancer Network (NCCN) guidelines, adjuvant ET for 5, and up to 10 years, is recommended for ER+ postmenopausal women to reduce the risk of relapse and breast cancer-related mortality.^3,4^ Among the estimated 268,600 new breast cancers diagnosed annually in North America, approximately one-third are in women aged 70 years and older.^5^ Statistical trends indicate a rising BC incidence in this age group. According to the Surveillance Epidemiology and End Results (SEER) database, patients aged ≥70 years may be as high as 40% of all breast cancer patients.^6^ Majority of early-stage invasive BC is estrogen-receptor positive (ER+).^7,8^ Breast cancer in older women is shown to be more indolent compared with the younger women.^9^

The commonly prescribed adjuvant ET drugs include tamoxifen (Tam) and aromatase inhibitors (AI), including anastrozole, letrozole, and exemestane.^10–12^ Published data have summarized the anticipated side effects, rates of compliance, and also the poor tolerance of ET among women with a history of comorbidities.^4,13,14^ BC in older (aged ≥70 years) postmenopausal women represents a distinct segment of the breast cancer population that are commonly underrepresented in most adjuvant therapy clinical trials.^15^ Accordingly, the magnitude of risk-benefit of treatment observed in younger women may not be directly translatable to the older BC patient population.^16^ In this understudied patient population balancing the tradeoffs between disease outcomes and the negative effect of endocrine therapies on HRQoL is not well understood.

There are limited data in the literature reporting on the impact on HRQoL by the type of adjuvant ET in the older postmenopausal population.^17–21^ Notably, published reports largely include younger postmenopausal population, with mean age range from 59.5 to 63.2.^20,21^ Only 26% of participants were 70 years and older in the Team Trial.^22^ The primary goal of this report is to evaluate the impact of Tam and AI in older women ≥70 years with early-stage ER-positive BC who underwent BCS and adjuvant RT, with ET as the only prescribed systemic therapy.

## Patients and Methods

**Fig. 1:**
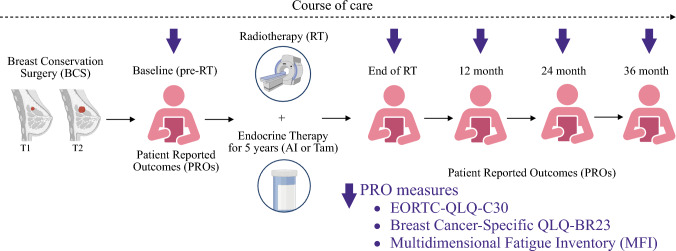
REQUITE study overview

The REQUITE group study (www.requite.eu) is a multicenter prospective study conducted across 26 countries in Europe and North America.^23^ The study was designed to identify and validate predictive genetic markers that predict risk of late toxicity following radiotherapy among breast, prostate, or lung cancer patients. A total of 4438 patients enrolled between April 2014 and October 2016. Among the 2057 BC patients, we identified 1052 women diagnosed with early-stage ER+ BC and treated with BCS and adjuvant RT and ET as the only prescribed systemic therapy. Among the 1052 cohort of patients, 201 were ≥70 years (Fig. 1) and eligible for our study.

The patient-reported outcomes (PRO) instruments used in this study include the validated European Organization for Research and Treatment of Cancer Quality of Life Questionnaire (EORTC QLQ)-C30, the Breast Cancer-Specific Quality of Life Questionnaire (BR23), and Multidimensional Fatigue Inventory (MFI). The EORTC QLQ-C30 measures five functional domains (physical, role, cognitive, emotional, and social), a global health status/QoL scale, three symptom scales (fatigue, pain, nausea and vomiting), and six single items assessing additional symptoms (dyspnea, appetite loss, sleep disturbance, constipation, diarrhea, and financial impact). The EORTC QLQ-BR23 specific for breast cancer patients to assess functional scales of body image, future perspectives, sexual functioning and sexual enjoyment, as well as symptom scales of breast and arm symptoms, systemic therapy side effects therapy, and hair loss. The MFI is a 20-item scale designed to evaluate five dimensions of fatigue (general fatigue, physical fatigue, reduced motivation, reduced activity, and mental fatigue).

In the REQUITE data set, the PRO measures were obtained at baseline after BCS, post-RT, and at 1, 2, and 3 years follow-up. Patient responses for each domain were obtained on a Likert scale. The PRO scores were calculated by normalizing raw scores to a scale ranging from 0 to 100 for simplifying interpretation purposes. Standardized scores, obtained through linear transformation, allow for the assessment of a patient's quality of life or functioning in different domains. Higher scores generally indicate better well-being, whereas lower scores suggest more symptoms or reduced functioning.

To address potential confounding factors stemming from any imbalance in PRO-related baseline characteristics, we employed propensity scores. These scores were computed for each patient via logistic regression, using baseline covariates, such as medications taken, body mass index, smoking history, alcohol intake, number of comorbidities, tumor histology, path T-stage, AI use, and Tam use as predictors. We also computed scores including the above covariates and 189 patients with known path *N*-stage.

### Statistical Methods

Data collection occurred at five timepoints: baseline (pre-RT), post-RT, 12 months post-RT, 24 months post-RT, and 36 months post-RT. Our primary outcomes of interest encompassed the mean PRO scores at each timepoint, the mean changes in PRO scores from baseline at each follow-up time within each age group, and the differences in mean changes from baseline, between age groups for each PRO domain and symptom score.

For the resulting propensity scores, we calculated the inverse probability of treatment weights (IPTW) for the average treatment effect among treated (ATT). These IPTW-ATT scores were subsequently integrated into the mixed model analysis of variance.

The mixed model analysis of variance, weighted by the ATT-calculated propensity scores, was employed to estimate means and mean changes from baseline over time within each age group. Moreover, it facilitated the comparison of these changes between the age groups. Our model featured a random intercept and an unstructured covariance matrix, effectively handling the correlated nature of observations within patients across multiple time points. It incorporated fixed effects for age group, time assessment, and their interaction, whereas covariate adjustment was accomplished by using the propensity score weights.

We conducted sensitivity analyses, including a multivariable mixed model analysis of variance (ANOVA) to assess the robustness of our IPTW-ATT analysis results. These analyses corroborated the estimands presented in the manuscript and bolstered the overall validity of our findings. All statistical analyses were conducted by using SAS Version 9.4, and hypothesis testing was performed at the 5% significance level.

## Results

**Fig. 2:**
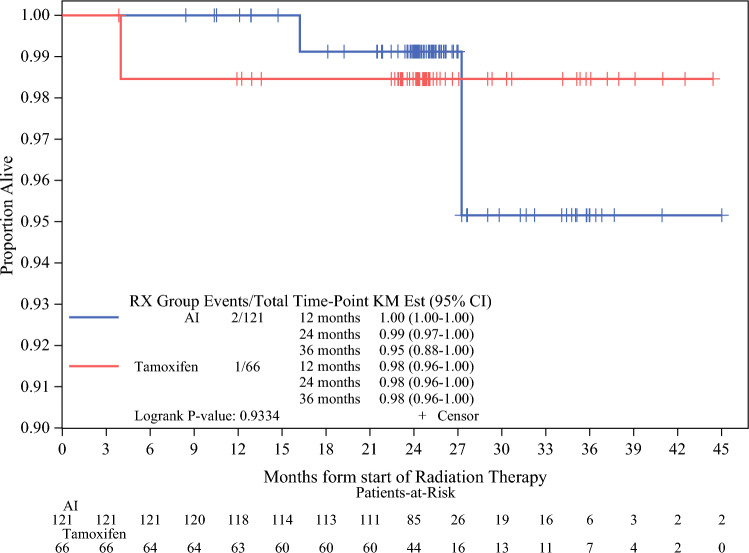
Kaplan-Meier plot for overall survival with the 95% confidence interval according to type of endocrine therapy

Among the 201 woman, 131 (65%) received AI and 70 (35%) received Tam. The mean age of the study cohort is 75.3 years. Table 1 summarizes patient demographics and medical profile. Of note, there were no differences between baseline incidence of two or more comorbidities and polypharmacy between the Tam and the AI groups. Women treated with Tam had significantly lower household income (*p* = 0.0446) and lower levels of education (*p* = 0.0183). The Tam group had significantly more favorable pathological features when compared to the AI group: smaller T-size (T1: 64% vs. 77%; *p* = 0.0057), and lower grade (Grade l: 26% vs. 16%; *p* = 0.0065) tumors. Treatment modalities also differed significantly between the groups. The Tam group had higher incidence of negative nodes (84.3% vs. 79.4%, *p* = 0.0068) compared with those in the AI group, and RT was more likely to be delivered using Intensity-Modulated Radiation Therapy (IMRT) (80% vs. 43%, *p* < 0.0001). Figure 2 shows the overall 3-year survival was similar in both treatment groups (*p* = 0.9334).

**Table 1:** Total patient demographics, clinicopathological, treatment characteristics according to type of endocrine therapy at diagnosis

	Aromatase inhibitor	Tamoxifen	Total	*p*
	(N = 131)	(N = 70)	(N = 201)	
Age, mean (SD)	75.4 (4.29)	75.3 (4.41)	75.3 (4.32)	0.8271
Body mass index, mean (SD)	27.8 (5.84)	26.4 (4.53)	27.3 (5.46)	0.1159
BMI, *n* (%)				0.2159
Underweight	3 (2.3%)	4 (5.7%)	7 (3.5%)	
Normal	41 (31.3%)	28 (40.0%)	69 (34.3%)	
Overweight/obese	87 (66.4%)	38 (54.2%)	125 (62.1%)	
Ethnicity, *n* (%)				0.1916
White	125 (95.4%)	70 (100.0%)	195 (97.0%)	
Other	6 (4.6%)	0 (0.0%)	6 (3.0%)	
Household income (per. month), *n* (%)				0.0446
Less than 3000	56 (78.9%)	40 (93.0%)	96 (84.2%)	
3000–<6000	15 (21.1%)	3 (7.0%)	18 (15.8%)	
Smoker, *n* (%)				0.9430
Never	94 (72.3%)	50 (73.5%)	144 (72.7%)	
Previous/current	36 (27.7%)	18 (26.5%)	54 (27.2%)	
Alcohol intake, *n* (%)				0.0775
Never	70 (54.3%)	25 (37.3%)	95 (48.5%)	
Previous/current	60 (45.7%)	42 (62.7%)	101 (51.5%)	
Education, *n* (%)				0.0183
Primary school	28 (27.2%)	24 (37.5%)	52 (31.1%)	
Secondary school	28 (27.2%)	25 (39.1%)	53 (31.7%)	
Professional school	29 (28.2%)	6 (9.4%)	35 (21.0%)	
University	18 (17.5%)	9 (14.1%)	27 (16.2%)	
Polypharmacy, *n* (%)	55 (42.0%)	21 (30.0%)	76 (37.8%)	0.0950
Two or more comorbidities, *n* (%)	59 (45.0%)	24 (34.3%)	83 (41.3%)	0.3272
Diabetes, *n* (%)	16 (12.2%)	6 (8.6%)	22 (10.9%)	0.4307
History of heart disease, *n* (%)	20 (15.3%)	11 (15.7%)	31 (15.4%)	0.93342
Rheumatoid arthritis, *n* (%)	8 (6.1%)	5 (7.1%)	13 (6.5%)	0.7760
Hypertension, *n* (%)	69 (52.7%)	40 (57.1%)	109 (54.2%)	0.54442
Depression, *n* (%)	17 (13.0%)	7 (10.0%)	24 (11.9%)	0.5352
Tumor histological type, *n* (%)				0.3157
Infiltrating ductal	84 (64.1%)	45 (64.3%)	129 (64.2%)	
Infiltrating lobular	25 (19.1%)	12 (17.1%)	37 (18.4%)	
Other	22 (16.8%)	13 (18.4%)	35 (18.4%)	
Path T stage, *n* (%)				0.0057
T1	84 (64.1%)	54 (77.1%)	138 (68.7%)	
T2 or greater	47 (35.9%)	10 (22.8%)	63 (31.4%)	
Path N stage, *n* (%)				0.0068
N negative	104 (79.4%)	59 (84.3%)	163 (81.1%)	
N positive	26 (19.9%)	5 (7.1%)	31 (15.4%)	
Tumor histological grade, *n* (%)				0.0065
Well	21 (16.2%)	18 (25.7%)	39 (19.5%)	
Moderate	80 (61.5%)	48 (68.6%)	128 (64.0%)	
Poor	29 (22.3%)	4 (5.7%)	33 (16.5%)	
Pathological tumor size (mm), median (Range)	17.0 (1.0, 52.0)	14.0 (2.0, 128.0)	16.0 (1.0, 128.0)	0.0459
Radiotherapy breast dose	40.5 (40.1, 50.0)	40.1 (40.1, 42.6)	40.1 (40.1, 45.0)	0.0482
Radiotherapy—no. fractions, median (IQR)	15.0 (15.0, 25.0)	15.0 (15.0, 16.0)	15.0 (15.0, 16.0)	0.1270
Radiotherapy—IMRT, *n* (%)	56 (42.7%)	56 (80.0%)	112 (55.7%)	<0.0001^1^
Radiotherapy—3D, *n* (%)	111 (85.4%)	54 (77.1%)	165 (82.5%)	0.1434
Radiotherapy—boost, *n* (%)	66 (50.4%)	35 (50.0%)	101 (50.2%)	0.9589
Axillary surgery, *n* (%)	130 (99.2%)	65 (92.9%)	195 (97.0%)	0.0113
Post operative infection, *n* (%)	12 (9.4%)	5 (7.5%)	17 (8.8%)	0.6418
Delayed healing, *n* (%)	4 (3.1%)	0 (0.0%)	4 (2.1%)	0.1421

### QLQ C30 Differences in Function

**Fig. 3:**
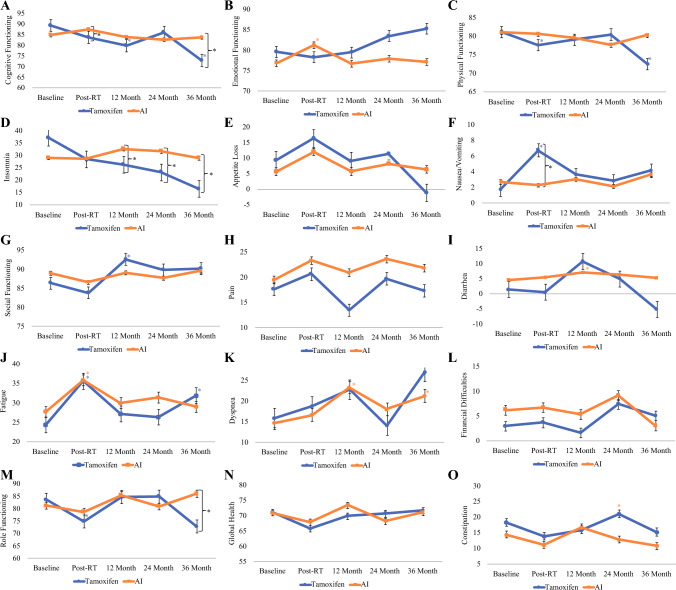
Mean EORTC30 scores for different symptoms in each age at different timepoints showing distribution of QoL outcomes (**A**–**O**) at baseline, end of radiotherapy (post-RT), 12, 24, and 36 months after radiotherapy. Error bars represent standard error. *p < 0.05

Notable differences were observed in cognitive functioning, emotional functioning, and physical functioning (Table 2). In the domain of cognitive functioning, women receiving AI showed a nonsignificant improvement (Δ2.62; *p* = 0.2822), whereas those receiving Tam exhibited a decline from baseline to post-RT (Δ-5.65; *p* = 0.0678). In the Tam group, the decline from baseline persisted throughout the follow-up period, with a decrease of Δ-9.44 points (*p* = 0.0347) at 12 months, Δ-3.23 points (*p* = 0.1944) at 24 months, and Δ-16.48 points (*p* = 0.0025) at 36 months. In contrast, the AI group experienced only a slight, nonsignificant decline in cognitive functioning from baseline levels at 12 (Δ-1.02; *p* = 0.6303), 24 (Δ-2.23; *p* = 0.2612), and 36 (Δ-1.17; *p* = 0.6962) months, suggesting a more pronounced negative impact of Tam on cognitive functioning over the follow-up duration of the study (Fig. 3A).

**Table 2:** Propensity score adjusted EORTC QLQ-C30, BR23, MFI scores over time by type of endocrine therapy

		Tamoxifen (Tam)		Aromatase inhibitor (AI)		Tamoxifen versus AI	
		Adjusted mean change from baseline [95% CI]	*p*	Adjusted mean change from baseline [95% CI]	*p*	Adjusted difference in mean change [95% CI]	*p*
Global health/QoL	Baseline	Reference		Reference			
	Post-RT	−5.28 [−9.42, −1.13]	0.0127	−3.12 [−6.8, 0.57]	0.0974	−2.16 [−7.71, 3.39]	0.4448
	12 Month	−1.15 [−4.83, 2.53]	0.5396	2.46 [−1.34, 6.26]	0.2043	−3.61 [−8.9, 1.68]	0.1809
	24 Month	−0.36 [−8.11, 7.39]	0.9273	−2.75 [−7.04, 1.54]	0.2089	2.39 [−6.47, 11.25]	0.5966
	36 Month	0.6 [−9.77, 10.97]	0.9096	0.08 [−6.91, 7.07]	0.9821	0.52 [−11.99, 13.03]	0.9350
Emotional functioning	Baseline	Reference		Reference			
	Post-RT	−1.29 [−9.05, 6.48]	0.7452	4.41 [0.79, 8.03]	0.0171	−5.7 [−14.27, 2.87]	0.1922
	12 Month	−0.13 [−9.12, 8.87]	0.9779	−0.07 [−4.01, 3.87]	0.9717	−0.06 [−9.88, 9.77]	0.9911
	24 Month	3.88 [−2.65, 10.41]	0.2437	1.1 [−3.74, 5.94]	0.6560	2.78 [−5.35, 10.91]	0.5016
	36 Month	5.67 [−3.72, 15.05]	0.2364	0.33 [−6.77, 7.42]	0.9275	5.34 [−6.43, 17.11]	0.3734
Social functioning	Baseline	Reference		Reference			
	Post-RT	−2.52 [−6.26, 1.23]	0.1875	−2.26 [−6.37, 1.86]	0.2822	−0.26 [−5.82, 5.31]	0.9271
	12 Month	6.27 [0.73, 11.8]	0.0266	0.19 [−4.01, 4.39]	0.9290	6.08 [−0.87, 13.02]	0.0864
	24 Month	3.49 [−7.71, 14.69]	0.5406	−1.29 [−6.06, 3.48]	0.5958	4.78 [−7.4, 16.96]	0.4409
	36 Month	3.86 [−2.79, 10.51]	0.2551	0.63 [−5.51, 6.76]	0.8415	3.23 [−5.82, 12.28]	0.4834
Cognitive functioning	Baseline	Reference		Reference			
	Post-RT	−5.65 [−11.72, 0.41]	0.0678	2.62 [−1.36, 6.6]	0.1970	−8.27 [−15.53, −1.01]	0.0256
	12 Month	−9.44 [−18.2, −0.68]	0.0347	−1.02 [−5.16, 3.13]	0.6303	−8.42 [−18.11, 1.27]	0.0884
	24 Month	−3.23 [−8.12, 1.65]	0.1944	−2.23 [−6.11, 1.66]	0.2612	−1.01 [−7.25, 5.24]	0.7516
	36 Month	−16.48 [−27.15, −5.81]	0.0025	−1.17 [−7.07, 4.73]	0.6962	−15.31 [−27.5, −3.11]	0.0140
Role functioning	Baseline	Reference		Reference			
	Post-RT	−8.77 [-14.55, -3]	0.0030	-2.58 [-6.6, 1.43]	0.2065	−6.19 [−13.22, 0.84]	0.0844
	12 Month	1.04 [-5.82, 7.9]	0.7658	4.23 [-0.58, 9.04]	0.0849	−3.19 [−11.57, 5.19]	0.4548
	24 Month	1.25 [-9.62, 12.13]	0.8209	-0.4 [-5.58, 4.77]	0.8780	1.66 [−10.39, 13.71]	0.7869
	36 Month	−10.82 [-24.9, 3.26]	0.1317	4.79 [-1.19, 10.76]	0.1161	−15.61 [−30.9, −0.31]	0.0455
Physical functioning	Baseline	Reference		Reference			
	Post-RT	−3.56 [−6.26, −0.85]	0.0100	−0.48 [−2.74, 1.79]	0.6798	−3.08 [−6.61, 0.45]	0.0870
	12 Month	−2.1 [−4.53, 0.33]	0.0903	−1.47 [−4.59, 1.66]	0.3564	−0.63 [−4.59, 3.33]	0.7545
	24 Month	−0.7 [−7.86, 6.46]	0.8485	−3.38 [−6.69, −0.07]	0.0456	2.68 [−5.21, 10.57]	0.5048
	36 Month	−8.64 [−15.26, −2.01]	0.0107	-0.82 [-5.93, 4.29]	0.7529	−7.82 [−16.18, 0.55]	0.0671
Fatigue	Baseline	Reference		Reference			
	Post-RT	11.2 [4.47, 17.94]	0.0012	8.11 [4.61, 11.61]	0.0000	3.1 [−4.49, 10.69]	0.4233
	12 Month	2.75 [−3.96, 9.46]	0.4205	2.24 [−2.07, 6.55]	0.3075	0.51 [−7.46, 8.49]	0.8995
	24 Month	1.99 [−3.98, 7.96]	0.5133	3.73 [−1.07, 8.53]	0.1274	−1.74 [−9.4, 5.92]	0.6549
	36 Month	7.51 [0.58, 14.44]	0.0337	1.26 [-6.16, 8.69]	0.7379	6.25 [-3.91, 16.4]	0.2274
Nausea/vomiting	Baseline	Reference		Reference			
	Post-RT	5.06 [0.16, 9.95]	0.0429	−0.38 [−2.47, 1.71]	0.7213	5.44 [0.11, 10.76]	0.0453
	12 Month	1.93 [−1.4, 5.25]	0.2563	0.35 [−1.84, 2.54]	0.7530	1.57 [−2.41, 5.56]	0.4380
	24 Month	1.09 [−1.57, 3.75]	0.4211	−0.56 [−2.96, 1.84]	0.6477	1.65 [−1.94, 5.23]	0.3664
	36 Month	2.42 [−2.77, 7.6]	0.3599	0.94 [−4.01, 5.9]	0.7087	1.47 [−5.69, 8.64]	0.6863
Pain	Baseline	Reference		Reference			
	Post-RT	2.99 [−3.29, 9.28]	0.3495	3.86 [−0.49, 8.21]	0.0818	−0.87 [−8.51, 6.78]	0.8240
	12 Month	−4.18 [−11.15, 2.79]	0.2392	1.47 [−3.14, 6.07]	0.5320	−5.65 [−14, 2.71]	0.1848
	24 Month	2.07 [−8.4, 12.54]	0.6975	4.12 [−1.46, 9.71]	0.1475	−2.05 [−13.92, 9.82]	0.7344
	36 Month	−0.3 [−9.35, 8.75]	0.9483	2.3 [−4.55, 9.15]	0.5101	−2.6 [−13.94, 8.75]	0.6532
Dyspnea	Baseline	Reference		Reference			
	Post-RT	2.82 [−8.53, 14.16]	0.6257	1.92 [−1.76, 5.59]	0.3057	0.9 [−11.03, 12.83]	0.8822
	12 Month	6.91 [−0.69, 14.5]	0.0748	8.6 [3.76, 13.44]	0.0005	−1.7 [−10.7, 7.31]	0.7117
	24 Month	−1.82 [−9.48, 5.83]	0.6403	3.35 [−1.71, 8.41]	0.1934	−5.18 [−14.35, 4]	0.2683
	36 Month	11.09 [−6.07, 28.25]	0.2046	6.6 [0.16, 13.03]	0.0444	4.5 [−13.83, 22.82]	0.6299
Insomnia	Baseline	Reference		Reference			
	Post-RT	−8.67 [−19.89, 2.55]	0.1298	−0.38 [−4.95, 4.2]	0.8718	−8.29 [−20.41, 3.83]	0.1795
	12 Month	−10.86 [−21.81, 0.1]	0.0521	3.54 [−1.55, 8.62]	0.1726	−14.39 [−26.47, −2.31]	0.0196
	24 Month	−13.86 [−25.76, −1.96]	0.0225	2.57 [−3.45, 8.58]	0.4024	−16.42 [−29.76, −3.09]	0.0159
	36 Month	−20.5 [−32.65, −8.36]	0.0010	−0.07 [−8.29, 8.15]	0.9864	−20.43 [−35.1, −5.77]	0.0064
Appetite loss	Baseline	Reference		Reference			
	Post-RT	6.97 [−3.1, 17.04]	0.1745	6.32 [2.61, 10.04]	0.0009	0.64 [−10.09, 11.37]	0.9062
	12 Month	−0.34 [−6.16, 5.48]	0.9081	0.01 [−3.09, 3.1]	0.9973	−0.35 [−6.94, 6.24]	0.9175
	24 Month	2.03 [−5.78, 9.85]	0.6092	2.57 [−0.97, 6.11]	0.1546	−0.53 [−9.11, 8.05]	0.9029
	36 Month	−10.5 [−21, 0.01]	0.0502	0.76 [−5.55, 7.08]	0.8126	−11.26 [−23.52, 1]	0.0717
Constipation	Baseline	Reference		Reference			
	Post-RT	−4.45 [-11.77, 2.86]	0.2320	−3.3 [−8.13, 1.53]	0.1804	−1.16 [−9.92, 7.61]	0.7953
	12 Month	−2.43 [-11.32, 6.46]	0.5916	2.28 [−2.03, 6.6]	0.2991	−4.71 [−14.6, 5.17]	0.3492
	24 Month	2.71 [−4.53, 9.94]	0.4626	−1.57 [−7.47, 4.33]	0.6010	4.28 [−5.06, 13.61]	0.3684
	36 Month	−3.17 [−16.28, 9.94]	0.6350	−3.62 [−10.42, 3.18]	0.2957	0.45 [−14.32, 15.22]	0.9522
Diarrhea	Baseline	Reference		Reference			
	Post-RT	−0.86 [−6.41, 4.68]	0.7599	0.72 [−1.62, 3.06]	0.5448	-1.58 [−7.6, 4.43]	0.6053
	12 Month	9.34 [−0.75, 19.43]	0.0695	2.44 [0.22, 4.67]	0.0314	06.9 [−3.43, 17.22]	0.1903
	24 Month	3.53 [−2.82, 9.88]	0.2755	1.64 [−0.77, 4.05]	0.1826	1.89 [-4.9, 8.68]	0.5845
	36 Month	−6.49 [−23.23, 10.24]	0.4463	0.59 [−3.29, 4.47]	0.7645	−7.09 [-24.27, 10.1]	0.4182
Financial difficulties	Baseline	Reference		Reference			
	Post-RT	0.72 [−3.15, 4.59]	0.7156	0.5 [−2.62, 3.61]	0.7537	0.22 [-4.75, 5.19]	0.9307
	12 Month	−1.35 [−6.53, 3.84]	0.6101	−0.89 [−4.21, 2.44]	0.6006	−0.46 [−6.62, 5.7]	0.8831
	24 Month	4.45 [−9, 17.9]	0.5157	2.94 [−1.54, 7.41]	0.1980	1.52 [−12.66, 15.69]	0.8336
	36 Month	2.09 [−5.7, 9.88]	0.5985	−3.19 [−8.36, 1.97]	0.2250	5.28 [−4.07, 14.63]	0.2674
BR 23 arm symptoms	Baseline	Reference		Reference			
	Post-RT	0.59 [−4.15, 5.32]	0.8078	−1.03 [−3.57, 1.5]	0.4234	1.62 [−3.75, 6.99]	0.5536
	12 Month	−3.55 [−8.51, 1.41]	0.1603	−0.35 [−3.95, 3.25]	0.8500	−3.2 [−9.33, 2.92]	0.3048
	24 Month	0.14 [−5.22, 5.49]	0.9602	0.72 [−2.72, 4.15]	0.6812	−0.58 [−6.94, 5.78]	0.8572
	36 Month	−7.5 [−16.61, 1.62]	0.1068	5.28 [−2.32, 12.88]	0.1728	−12.78 [−24.65, −0.91]	0.0349
BR 23 body image	Baseline	Reference		Reference			
	Post-RT	−1.06 [−4.05, 1.94]	0.4884	−0.93 [−3.85, 1.98]	0.5302	−0.13 [−4.3, 4.05]	0.9528
	12 Month	−2.53 [−9.81, 4.75]	0.4951	−0.45 [−3.7, 2.79]	0.7833	−2.07 [−10.04, 5.9]	0.6093
	24 Month	3.73 [−2.04, 9.5]	0.2043	−2.24 [−5.24, 0.76]	0.1431	5.97 [−0.53, 12.48]	0.0718
	36 Month	3.76 [−4, 11.52]	0.3418	−1.34 [−6.23, 3.55]	0.5894	5.1 [−4.07, 14.28]	0.2749
BR 23 future perspective	Baseline	Reference		Reference			
	Post-RT	1.5 [−5.14, 8.15]	0.6565	2.44 [−1.82, 6.71]	0.2606	−0.94 [−8.83, 6.96]	0.8154
	12 Month	5.48 [−1.77, 12.74]	0.1382	4.54 [−1.06, 10.13]	0.1116	0.95 [−8.21, 10.11]	0.8390
	24 Month	9.02 [−5.03, 23.06]	0.2078	0.6 [−4.94, 6.13]	0.8316	8.42 [−6.68, 23.51]	0.2739
	36 Month	12.01 [−2.09, 26.11]	0.0950	6.2 [−4.01, 16.42]	0.2334	5.8 [−11.61, 23.22]	0.5128
BR 23 upset by hair loss	Baseline	Reference		Reference			
	Post-RT	−4.53 [−17.28, 8.22]	0.4824	4.25 [−5.8, 14.3]	0.4027	−8.78 [−25.01, 7.46]	0.2855
	12 Month	11.47 [−10.55, 33.48]	0.3034	7.2 [−7.85, 22.26]	0.3444	4.27 [−22.4, 30.94]	0.7513
	24 Month	−1.61 [−17.4, 14.18]	0.8397	1.54 [−13.66, 16.74]	0.8412	−3.15 [−25.07, 18.77]	0.7759
	36 Month	6.28 [−25.74, 38.29]	0.6977	0.25 [−20.73, 21.22]	0.9815	6.03 [−32.24, 44.31]	0.7548
BR 23 sexual enjoyment	Baseline	Reference		Reference			
	Post-RT	−0.79 [−15.5, 13.91]	0.9138	−2.31 [−13.29, 8.66]	0.6733	1.52 [−16.83, 19.87]	0.8684
	12 Month	2 [−14.19, 18.18]	0.8048	−6.15 [−19.64, 7.34]	0.3633	8.15 [−12.92, 29.21]	0.4400
	24 Month	−5.2 [−49.18, 38.78]	0.8128	−10.72 [−30.95, 9.5]	0.2911	5.53 [−42.88, 53.93]	0.8191
	36 Month	−30.08 [−52.23, −7.93]	0.0089	18.41 [−2.4, 39.22]	0.0815	−48.49 [−78.89, −18.1]	0.0024
BR 23 sexual functioning	Baseline	Reference		Reference			
	Post-RT	−2.1 [−5.04, 0.84]	0.1605	−0.66 [−2.9, 1.58]	0.5631	−1.44 [−5.14, 2.25]	0.4426
	12 Month	−3.73 [−7.69, 0.23]	0.0650	−1.1 [−3.83, 1.63]	0.4281	−2.63 [−7.44, 2.18]	0.2831
	24 Month	−2.7 [−6.72, 1.31]	0.1863	−0.02 [−3.61, 3.57]	0.9916	−2.69 [−8.07, 2.7]	0.3277
	36 Month	−7.07 [−13.14, −1]	0.0226	−2.11 [−7.69, 3.46]	0.4570	−4.96 [−13.2, 3.29]	0.2378
BR 23 systemic therapy side effects	Baseline	Reference		Reference			
	Post-RT	4.66 [−1.16, 10.47]	0.1162	3.15 [1.03, 5.27]	0.0037	1.51 [−4.68, 7.69]	0.6329
	12 Month	4.27 [0.02, 8.53]	0.0489	5.03 [2.73, 7.34]	0.0000	−0.76 [−5.6, 4.08]	0.7578
	24 Month	4.99 [1.95, 8.03]	0.0014	5.6 [2.89, 8.31]	0.0001	−0.61 [−4.69, 3.46]	0.7683
	36 Month	6.75 [0.96, 12.55]	0.0225	5.38 [0.62, 10.14]	0.0267	1.37 [−6.13, 8.87]	0.7199
BR 23 breast symptoms	Baseline	Reference		Reference			
	Post-RT	13.94 [7.07, 20.82]	0.0001	11.39 [7.79, 14.99]	0.0000	2.56 [−5.2, 10.32]	0.5172
	12 Month	−2.1 [−13.38, 9.18]	0.7142	−0.27 [−4.07, 3.54]	0.8900	−1.84 [−13.74, 10.07]	0.7621
	24 Month	−7.19 [−13.97, −0.41]	0.0377	−6.57 [−9.84, −3.29]	0.0001	−0.62 [−8.15, 6.91]	0.8709
	36 Month	−2.64 [−12.91, 7.64]	0.6143	−6.43 [−13.05, 0.19]	0.0569	3.79 [−8.43, 16.02]	0.5422
General fatigue	Baseline	Reference		Reference			
	Post-RT	2.18 [0.72, 3.64]	0.0036	1.21 [0.42, 2.01]	0.0029	0.97 [−0.69, 2.63]	0.2522
	12 Month	1.72 [0.17, 3.27]	0.0302	0.14 [−0.72, 1]	0.7508	1.58 [−0.19, 3.36]	0.0806
	24 Month	1.04 [−0.32, 2.39]	0.1322	0.43 [−0.69, 1.56]	0.4494	0.6 [−1.16, 2.37]	0.5010
	36 Month	−1.95 [−2.85, −1.05]	0.0000	0.09 [−1.18, 1.36]	0.8858	−2.04 [−3.59, −0.49]	0.0102
Physical fatigue	Baseline	Reference		Reference		Reference	
	Post-RT	1.91 [0.06, 3.75]	0.0428	1.38 [0.61, 2.14]	0.0004	0.53 [−1.47, 2.52]	0.6046
	12 Month	0.74 [−0.64, 2.11]	0.2933	0.1 [−0.73, 0.93]	0.8108	0.63 [−0.97, 2.24]	0.4381
	24 Month	0.37 [−1.43, 2.17]	0.6848	0.36 [−0.59, 1.31]	0.4548	0.01 [−2.02, 2.05]	0.9911
	36 Month	−3.09 [−4.07, −2.12]	0.0000	0.13 [−1.41, 1.66]	0.8713	−3.22 [−5.04, −1.4]	0.0006
Reduced activity	Baseline	Reference		Reference		Reference	
	Post-RT	1.27 [−0.07, 2.61]	0.0636	1.45 [0.66, 2.24]	0.0004	−0.18 [−1.74, 1.38]	0.8211
	12 Month	0.39 [−1.33, 2.11]	0.6586	−0.08 [−0.77, 0.61]	0.8177	0.47 [−1.38, 2.32]	0.6200
	24 Month	0.33 [−0.99, 1.66]	0.6194	0.26 [−0.56, 1.08]	0.5368	0.08 [−1.48, 1.63]	0.9224
	36 Month	−2.26 [−3.15, −1.37]	0.0000	−1.01 [−2.05, 0.02]	0.0551	−1.25 [−2.61, 0.12]	0.0730
Reduced motivation	Baseline	Reference		Reference		Reference	
	Post-RT	2.65 [1.19, 4.12]	0.0004	0.92 [0.09, 1.75]	0.0299	1.74 [0.06, 3.42]	0.0429
	12 Month	2.05 [0.19, 3.91]	0.0305	−0.62 [−1.33, 0.09]	0.0880	2.67 [0.68, 4.66]	0.0087
	24 Month	1.43 [−0.05, 2.91]	0.0579	0.02 [−0.89, 0.94]	0.9575	1.4 [−0.33, 3.14]	0.1131
	36 Month	0.05 [−0.9, 1.01]	0.9112	−0.54 [−2.07, 0.98]	0.4831	0.6 [−1.2, 2.39]	0.5133
Mental fatigue	Baseline	Reference		Reference		Reference	
	Post-RT	1.16 [−0.99, 3.3]	0.2893	0.28 [−0.63, 1.18]	0.5485	0.88 [−1.45, 3.21]	0.4568
	12 Month	0.83 [−0.84, 2.5]	0.3297	0.17 [−0.65, 0.99]	0.6866	0.66 [−1.2, 2.53]	0.4854
	24 Month	0.41 [−1.65, 2.48]	0.6935	0.83 [−0.08, 1.74]	0.0720	−0.42 [−2.67, 1.83]	0.7140
	36 Month	3.14 [1.89, 4.39]	0.0000	−0.76 [−2.13, 0.61]	0.2762	3.9 [2.05, 5.75]	0.0000

In the domain of emotional functioning, women receiving AI exhibited a transient but significant improvement from baseline to post-RT (Δ+4.41; *p* = 0.0171), followed by a return to baseline levels from post-RT to 36 months (Δ-4.08; *p* = 0.2424) (Fig. 3B). In contrast, women receiving Tam showed slightly reduced emotional functioning from baseline to post-RT (Δ-1.29; *p* = 0.7452) but demonstrated substantial steady improvement above baseline levels from post-RT to 36 months (Δ+6.95; *p* = 0.0975).

In the domain of physical functioning, women receiving AI showed no significant change from baseline to post-RT (Δ-0.48; *p* = 0.6798). However, there was no significant drop from post-RT to 24 months (Δ-2.90; *p* = 0.0873), followed by an improvement back to baseline functioning levels from 24 to 36 months (Δ+2.56; *p* = 0.2668) (Fig. 3C). In contrast, women receiving Tam exhibited a significant reduction in physical functioning from baseline to post-RT (Δ-3.56; *p* = 0.0100). Although functioning returned to baseline levels from post-RT to 24 months (Δ2.86; *p* = 0.4920), we observed a substantial drop in physical functioning of Δ-7.94 points at 36 months (*p* = 0.0495).

### QLQ C30 Differences in Symptoms

Patient symptoms differed between the two groups, with observed differences in nausea and vomiting, insomnia, and appetite loss over time (Table 2). Women receiving AI showed no change in symptoms of insomnia from baseline to post-RT (Δ-0.38; *p* = 0.8718), there was a transient worsening of insomnia symptoms at 12 months (Δ+3.91; *p* = 0.1794), before returning to baseline levels from 12 to 36 months (Δ-3.61; *p* = 0.3762). In contrast, women receiving Tam did not report symptoms of insomnia from post-RT to 12 months (Δ-2.19; *p* = 0.6980) and experienced sustained improvement between 12 to 36 months (Δ-9.65; *p* = 0.1509).

Both the AI and Tam groups experienced similar patterns of appetite loss from baseline to post-RT, followed by a return to baseline levels at 12 months. However, as illustrated in Fig. 3E, women receiving Tam showed significantly less appetite loss from 24 to 36 months (Δ-12.53; *p* = 0.0124) compared with those receiving AI (Δ-1.81; *p* = 0.5818; *p* = 0.0730).

As illustrated in Fig. 3F, the AI group showed no significant change in symptoms of nausea and vomiting from baseline to post-RT (Δ-0.38; *p* = 0.7213), from post-RT to 12 months (Δ+0.73; *p* = 0.4708), and from 12 to 36 months (Δ+0.59; *p* = 0.7716). Conversely, women in the Tam group experienced a significant increase in symptoms of nausea and vomiting from baseline to post-RT (Δ+5.06; *p* = 0.0429), followed by a notable decrease from post-RT to 12 months (Δ-3.13; *p* = 0.2302), and minimal additional change from 12 to 36 months (Δ+0.49; *p* = 0.8394).

### EORTC BR23 Measures

**Fig. 4:**
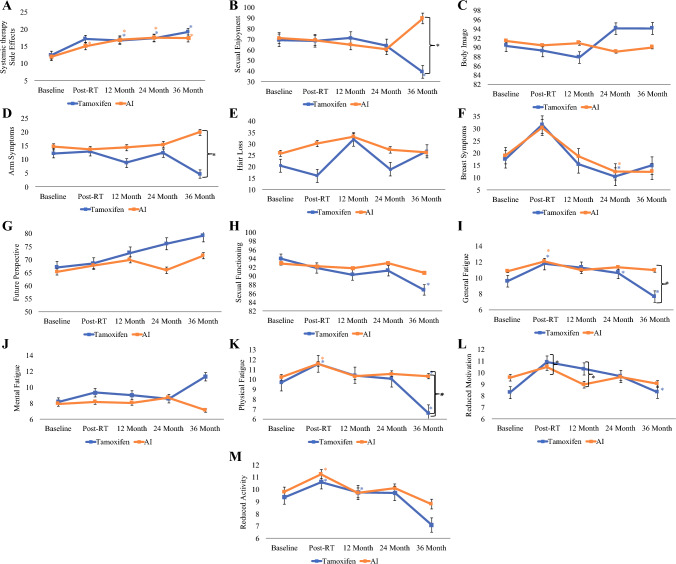
Mean scores for different symptoms in each age at different timepoints showing distribution of QoL outcomes for BR23 (**A–H**) and MFI20 (**I–M**) at baseline, end of radiotherapy (post-RT), 12, 24, and 36 months after radiotherapy. Error bars represent standard error. *p < 0.05

The pattern of systemic therapy side effects was similar for both the AI and Tam groups (Fig. 4A). Both groups demonstrated significant increases in systemic therapy side effects at 12, 24, and 36 months (Tam: *p* = 0.0489, 0.0014, 0.0225 and AI: *p* < 0.001, *p* = 0.001, *p* = 0.0267), respectively.

Notable differences were observed in BR23 sexual enjoyment between the treatment groups (Table 2). Trends in sexual enjoyment were quite similar for both groups until the 24 months follow-up, after which notable distinctions emerged. As illustrated in Fig. 4B, at 36 months, the Tam group experienced a large reduction (Δ-24.88; *p* = 0.0947), whereas the AI group showed a significant increase in enjoyment (Δ+29.14; *p* = 0.0153).

The AI group reported a slight drop in body image from baseline to post-RT (Δ-0.93; *p* = 0.5302), followed by an improvement back to baseline values from post-RT to 12 months (Δ+0.48; *p* = 0.7701), and with no significant change observed from 12 to 36 months (Δ-0.89; *p* = 0.7325). Conversely, as illustrated in Fig. 4C, the Tam group had a similar reduction from baseline to post-RT (Δ-1.06; *p* = 0.4884), followed by a nonsignificant drop from post-RT to 12 months (Δ-1.47; *p* = 0.7213), and then remarkable improvement from 12 to 36 months (Δ+6.29; *p* = 0.1873).

### MFI Observations

Notable differences were observed in MFI measures of general, physical, and mental fatigue between the treatment groups (Table 2). Trends in general fatigue were quite similar for both groups until the 24 month follow-up, after which notable distinctions emerged, as presented in Fig. 4I. At 36 months, the AI group showed almost no reduction in general fatigue (Δ-0.34; *p* = 0.5749), whereas the Tam group experienced a large reduction (Δ-2.99; *p* < 0.0001). Trends in mental fatigue were quite similar for both groups until the 24-month mark, after which notable distinctions emerged (Fig. 4J). At 36 months, the AI group showed a decrease in mental fatigue (Δ-1.59; *p* = 0.0103), whereas the Tam group experienced a large increase (Δ+2.73; *p* < 0.0001). Figure 4K illustrates trends in physical fatigue that were quite similar for both groups until the 24-month mark, after which notable distinctions emerged. At 36 months, the AI group showed almost no reduction in physical fatigue (Δ-0.23; *p* = 0.7543), whereas the Tam group experienced a large reduction (Δ-3.47; *p* < 0.0001).

We analyzed the data adjusted for known path N-stage (*n* = 189). The observations on QoL scores were not dissimilar from the observations of the entire cohort (*n* = 201).

## Discussion

The longitudinal effects of ET on HRQoL that have been described.^17–21^ Table 3 summarizes published literature and notes differential pattern on QoL by type of ET prescribed.^20–22^ The NSAS BC 03 trial comparing Tam with Anastrozole observed that among younger postmenopausal women, the Tam group had better FACT-B, FACT-G, and the FACT-ES scores compared with the anastrozole group (*p* = 0.042, 0.038, and 0.005, respectively). Results of the randomized National Surgical Adjuvant Study of Breast Cancer (N-SAS BC) trial on women aged ≥60 years, who had received definitive surgery for hormone receptor-positive BC and ET, reported significantly worse diarrhea and headache in the AI group compared with the Tam group.^20^ The NSAS BC 04 trial that includes a smaller number of patients (*n* = 166) compared Tamoxifen to exemestane and reported that the FACT-B scores improved after treatment began and remained significantly higher in the tamoxifen group than in the exemestane or anastrozole groups for 1 year (*p* = 0.045). The Tam Exemestane Adjuvant Multinational (TEAM) trial compared Tam to exemestane and reported that exemestane users experienced more insomnia compared with those taking Tam.^22^ Our study, focused exclusively on older postmenopausal BC patients who receive both endocrine therapy and radiation following breast conservation surgery, and our observations illustrate a differential impact of the type of ET on HRQoL. Findings from our study suggest that AI was associated with more symptoms of insomnia compared to Tam. Similar observations have been reported in TEAM trial. Based on the PROs, we note a greater decline in cognitive functioning over time within the Tam group, which raises concerns about potential cognitive side effects associated with Tam, particularly concerning in an older population already at risk for cognitive decline. In contrast, the AI group retained a relatively stable cognitive functioning, which may be a consideration for clinicians when determining treatment options.

**Table 3:** Summary of literature on the effect of type of endocrine therapy on QoL

Author	Endocrine therapy	Number of patients	Mean age, (SD or %)	QOL instruments	Observations reported
Ohsumi et al. Japan N-SAS BC 03	Tamoxifen vs. switching to Anastrozole after adjuvant Tamoxifen for 1–4 years	694	Tamoxifen: 60.2 (7.4) Anastrozole: 59.5 (7.4)	FACT-B, FACT- ES, CES-D, FACT- G	FACT-B, FACT-G, and FACT-ES total scores were statistically significantly better in the tamoxifen group than in the anastrozole group (*p* = 0.042, 0.038, and 0.005, respectively)
Takei et al. Japan N-SAS BC 04	Tamoxifen versus Exemestane versus Anastrozole	166	Tamoxifen: 63.0 (8.1) Anastrozole: 62.9 (8.0) Exemestane: 63.2 (6.9)	FACT-B, FACTES, CES-D, FACT-G	FACT-B scores increased after treatment began and remained significantly higher in the tamoxifen group than in the exemestane or anastrozole groups for one year (*p* = 0.045) In all patients assigned to exemestane or tamoxifen, FACT-B scores increased after treatment began and remained significantly higher in the tamoxifen group than in the exemestane group for one year (*p* = 0.047)
Van Nes et al. TEAM Trial	Tamoxifen versus Exemestane	543	<50–59 (37%) 60–69 (37%) ≥70 (26%)	EORTC QLQ-C30 and B23, FASCT-ES	Exemestane treated patients experienced more insomnia compared to Tamoxifen (*p* = 0.001)
Li et al. (present study)	Tamoxifen versus Anastrozole	201	Aromatase Inhibitor: 75.4 (4.29) Tamoxifen: 75.3 (4.41) [All women ≥70 years]	EORTC QLQ-C30, B23, and MFI	Patients treated with aromatase inhibitors experienced worse insomnia, and general and physical fatigue. Tamoxifen treated patients experienced more decline in physical and cognitive functioning compared with the aromatase inhibitor treated patients. The tamoxifen treated patients also reported more mental fatigue and reduced sexual enjoyment compared to the aromatase inhibitor treated patients.

In our study, notable differences in general and physical fatigue observed improved throughout the follow-up, whereas mental fatigue worsened in Tam compared with AI. These observations are similar to the findings from the Arimidex, Tamoxifen, Alone or in Combination (ATAC) trial and N-SAS BC substudy, and the TEAM trial.^21,24^ Among a Japanese postmenopausal patient population with hormone-sensitive BC, 16% and 5% of patients who received anastrozole or Tam reported fatigue, respectively.^21^

Publications on patient compliance to ET have reported that approximately 24% to 30% of patients discontinue AI owing to its toxic effects during the first 2 years.^25,26^ The negative impact of adjuvant therapies on the HRQoL of patients may contribute to high noncompliance rates. Our study could not evaluate the impact of HRQoL on adherence to ET, because the REQUITE dataset does not include data on treatment compliance. For improved understanding of the association of HRQoL and treatment compliance further prospective studies are needed. The other limitation of the present study is that observations reported are based on data collected from predominantly White patients. These may be different in women of other races/ethnic backgrounds.

Although our study is not a randomized trial, we use propensity matching to reduce the potential skewing of comparative outcomes. It encompasses prospectively collected PROs data on a large sample of uniformly treated older postmenopausal women followed over 3 years. Our study provides insight into the differential impact of type of ET on symptomatology and functioning across different domains of HRQoL with a focus on older women. In the multifaceted nature of QoL in this vulnerable population, these trends provide a foundation for developing patient-centered care strategies. This study adds to the growing literature on HRQoL in older patients with early-stage ER+ BC. Further research is needed to optimize the selection of risk-tailored adjuvant treatment options and to inform treatment decisions for older ER-positive BC patients, considering understanding of trade-offs between disease outcomes and HRQoL.

## Data Availability

Not applicable.
